# A. V. M. A. Echoes

**Published:** 1902-09

**Authors:** 


### A. V. M. A. ECHOES.

SEVERAL Eastern veterinarians on their return from the convention stopped for a day at Niagara Falls, and they tell strange stories of a visit to the "Burning Spring" on the Canadian side. The Canucks are not slow.

State Veterinarian Pearson, of Pennsylvania, not satisfied with the joyous opportunities afforded by our Minnesota colleagues, remained for several days after the convention to visit and inspect many other attractions of the "Gopher State."

Dr. W. L. Williams, of Ithaca, N. Y., was among those who joined the "JOURNAL Special" en route to Chicago.

New Jersey's coterie of prominent veterinarians, guided by Treasurer Lowe, of the American Veterinary Medical Association, after journeying to Chicago with the JOURNAL's special party, completed their journey via the Michigan Central. Drs. Smith, of Jersey City, and Glennon, of Newark, were among the number from the "Jersey Blue State."

At Buffalo the JOURNAL's special party was added to by Drs. William Dougherty, of Baltimore, Md., and J. C. Foelker, of Allentown, Pa.

At Sayre, New York State, Dr. C. J. Marshall and wife, of Philadelphia, Pa., joined the "JOURNAL Special."

Dr. D. E. Salmon, Chief of the Bureau of Animal Industry, started from Philadelphia, Pa., and journeyed to Minneapolis with the JOURNAL's party.

From far-away Arizona came that genial aggressive member of the profession and Territorial veterinarian, Dr. J. C. Norton, to join the "JOURNAL Special" at Chicago over the Burlington route to Minneapolis.

The Chicago special party over the Chicago, Milwaukee and St. Paul were so overfed at the sumptuous repast spread that they arrived some five hours late as a result of their somnolent indulgences.

The Canadian silver souvenir of the Chicago special party is said to have been indirectly an agent in delaying the arrival of the party, owing to soporific influences produced.

